# Alone but not isolated: social presence and cognitive load in learning with 360 virtual reality videos

**DOI:** 10.3389/fpsyg.2024.1305477

**Published:** 2024-06-28

**Authors:** Suhyun Ki, Sanghoon Park, Jeeheon Ryu, Jongho Kim, Inki Kim

**Affiliations:** ^1^Center for Immersive Learning Technology, Institute of Educational Research, Department of Education, Chonnam National University, Gwangju, Republic of Korea; ^2^Department of Educational and Psychological Studies, University of South Florida, Tampa, FL, United States; ^3^Department of Industrial and Enterprise Systems Engineering & Health Care Engineering Systems Center, University of Illinois Urbana-Champaign, Urbana, IL, United States

**Keywords:** 360 virtual reality, head mounted display, social presence, cognitive load, video lecture

## Abstract

**Introduction:**

This study aimed to identify any differences in social presence and cognitive load among three types of 360 virtual reality (VR)-based videos lectures. We hypothesized that social presence would be higher when interactions among peers are visible in a 360 VR video lectures while the cognitive load would be also increased.

**Methods:**

A total of 48 college students were randomly assigned to one of the three study groups to view an assigned 360 VR video lecture. The three groups were: (1) an instructor-only video viewing group, (2) a classroom lecture video viewing group, and (3) a classroom lecture and activity video viewing group. The video lectures were differently designed depending on the levels of peer visibility and the interactions between the instructor and peers. The participants watched one of the three types of assigned video lecture and subsequently completed two sets of questionnaires regarding social presence and cognitive load. A multivariate analysis of variance (MANOVA) was conducted with a planned contrast analysis for the type of video lectures.

**Results:**

We found that, contrary to the hypotheses, students in the group 1 (instructor-only video) showed higher social presence scores than students in the groups 2 and 3. However, no significant differences were found in the cognitive load scores.

**Discussion:**

The results show that 360 VR video lectures with an instructor-only are more effective at enhancing users’ social presence than 360 VR video lectures with both the instructor and class-peers. We suggest creating 360 VR video lectures with the presence of the course instructor to offer learners the sense of actually participating in a lecture.

## 1 Introduction

Although online video learning is becoming one of the most popular instructional methods, it is more difficult to create rich social environments for learners compared to face-to-face learning. Students have reported that when they perceive a sense of disconnection during online learning, their online experiences are less enjoyable, less helpful, and they experience more frustration than when they experience in-person interactions through their studies (Boling et al., 2012). Moreover, interactions with other people in learning environments have shown to be effective in helping learners organize their thoughts, reflect on their understanding, and identify gaps in their comprehension (Okita et al., 2012). Hence, video lectures delivered online need to be designed to enhance social connections and to provide various learner–system-interaction features (Breslow et al., 2013; Alraimi et al., 2015). Increasing interactions during online video learning to enhance social context would be essential.

To overcome the reported sense of disconnection, recent studies claimed that online video lectures need to consider improving sensory immersiveness to successfully engage students in the learning environment (Dede et al., 2017; Cesari et al., 2021). Especially, 360 virtual reality (VR) videos have been studied as an effective approach to add such immersiveness into online contents in higher education (Snelson and Hsu, 2020). 360 VR video lectures and its subsequent viewing using a VR headset together can imitate an authentic academic classroom setting, in which the students perceive a realistic scene in a lecture (Hebbel-Seeger et al., 2021). For this reason, 360 VR video lectures show potential as an effective educational tool (Lampropoulos et al., 2021) in that it is widely available (Shadiev et al., 2021), cost-effective (Ulrich et al., 2021), affordable, and easily accessible (Roche et al., 2021). Overall, 360 VR technology’s positive aspects significantly enhance learners’ immersive perception by effectively providing contextualized scenes during the learning process. This heightened immersiveness facilitates learners’ engagement and paves the way for a more interactive and dynamic learning experience. The result is a more engaged learning experience, which leads to higher learning achievement.

One of the strengths of 360 VR video lectures is to create a virtual learning environment that elicits a high degree of social presence (Cheng and Tsai, 2019; Araiza-Alba et al., 2021; Roche et al., 2021). Social presence refers to the degree to which a person is perceived as a “real person” in mediated communication (Gunawardena and Zittle, 1997, p.9). Social presence helps reduce learner’s feeling of isolation in online video learning (Borup et al., 2012) by offering visual representations of classroom and learning interactivity through the video (Oh et al., 2018). A 360 VR videos use real-world footage (Evens et al., 2022) with high visual-realism, can enhance both the immersive quality of the experience and the students’ perception of social presence. A 360 VR video also can capture complex learning interactions between the course instructor and students that can be replayed as many times as needed (Andel et al., 2020). Therefore, 360 VR-video users can perceive the virtual environment as an authentic learning experience (Southgate, 2018) that enhance to learning by replicating the real-world environment (Cheng and Tsai, 2019; Araiza-Alba et al., 2021). Furthermore, user engagement increases when the 360 VR video allows users to explore video details (Roche et al., 2021). The spatial display by 360 VR brings authentic context so that the user may have strong perceptions of social presence.

However, it should be noted that 360 VR videos do not always have a positive influence on perceiving social presence. Paradoxically, the high visual realism of 360 VR videos creates several limitations in terms of effective design of such VR videos. First, possibility for user-interactions within the 360 VR video are often minimal (Pirker and Dengel, 2021). Because 360 VR applications cannot provide direct user interaction with the content (Torres et al., 2020). From the previous study, although users can interact with the recording by freely choosing their viewing direction, the recorded event cannot be manipulated as programmed virtual reality scenarios allow (Roche et al., 2021). The experiences within 360 VR videos are often designed to provide a fixed camera angle. This experience only permits simple operations, such as turning around and changing viewpoints. The interaction from 360 VR is not strong enough to enhance the social presence.

Another possible limitation of 360 VR video is the unnecessary cognitive load caused by the amount of visual information when conveyed through the VR-headset. Although complex visual representations and details in 360 VR videos offer high representational fidelity and can lead to higher user-presence, they can also result in higher extraneous cognitive load which decreases learning (Makransky and Peterson, 2021). In 360 VR videos, the visual load increases as the learner adjusts screen details, which increases the extrinsic cognitive load, especially when the 360 VR video provides little visual guidance due to the wide field of view (Beege et al., 2012). Moreover, because 360 VR video users can navigate freely with little-to-no guidance, certain events can be easily overlooked (Ardisara and Fung, 2018), especially when users switch their focus between their main information-target and other details (Lin et al., 2017). Moreover, the transient information present in 360 VR videos can cause learners to miss learning objectives because of inherent limits of people’s working memory (Beege et al., 2023). Therefore, 360 VR videos require an appropriate design to lower the extraneous cognitive load while maintaining a high social presence.

Seeing other people who share or interact with the same virtual environment as the user increases social presence (Oh et al., 2018). The more prominent the person or character are to the user, the more they feel like they are sharing a space with him or her, even if there is no mutual recognition (Pimentel et al., 2021). For example, users can feel socially present by recognizing the presence of other learners in the virtual classroom. In addition, users can use the other learners to acquire their learning skills by mimicking how their peers learn, such as by asking questions. Even in cases where there are disagreements between the instructor and certain learners, this can increase user interaction and motivation to learn (Chi et al., 2017). However, even though the presence of peers in VR-learning videos can increase a user’s social presence (Garrison and Cleveland-Innes, 2004; Zhu et al., 2022), sharing the VR-environment with other learners can compete with certain cognitive learning process. One study found that the larger the number of learners in the same virtual space, the perception of the task’s difficulty increased, and the higher the cognitive load required to comprehend the learning content (Skuballa et al., 2019). It is necessary to compare the learning effects based on the presence and level of interaction with other peer learners when using VR videos to facilitate practical educational applications.

This study attempted to understand how the presence and interaction of instructor and peers in 360 VR videos influence students’ perception of social presence and cognitive load. Three types of 360 VR videos were created: (1) instructor-only, (2) classroom-lecture, and (3) classroom lecture and activity. Because these combinations created a virtual learning situation, their use can be assumed analogous to that of the role of an instructor. Online learning using the three video types was classified depending on peer-presence and peer-interaction. We conducted a preplanned comparison of the effects of the video types based on the presence or absence of peers in the videos; (1) versus (2) + (3). We also compared the effectiveness between video types with and without instructor–peer interaction and activity; (1) + (2) vs. (3). Therefore, the purpose of this study was to investigate the effects of three types of 360 VR videos on students’ perception of social presence and cognitive load.

### 1.1 Research questions and hypothesis

Two research questions guided this study as follows:

RQ1. Are there differences in social presence and cognitive load depending on the person featured (instructor-only vs. instructor and peers) in the 360 VR videos?

*H*1: Participants that view the 360 VR video that contains peers will have a higher social presence than those who viewed the instructor-only video.

*H*2: Participants who viewed the 360 VR video that contains peers will have a higher cognitive load than those who viewed the instructor-only video.

Justifications for hypotheses 1&2: RQ1 explores the difference between persons featured in 360 VR videos. Because it is assumed that educational videos are based in a classroom setting, it is natural for instructors or peers to appear in the video. In this study, we developed two types of videos, one featuring only the instructor and the other featuring both the instructor and peers. H1 and H2 are our hypotheses regarding the first research question. The presence or absence of characters in 360 videos can make a significant difference in social presence. The more prominent the characters are to the user, the more they feel like they are with them, even if there is no mutual recognition (Pimentel et al., 2021). In contrast, the more other students are present, the greater the number of distractions, which competes for finite working-memory resources, and the task is perceived by the viewer as being more difficult (Skuballa et al., 2019). It is crucial to identify how the presence of persons will have an impact on cognitive load to make a good balance for learning.

RQ2. Are there differences in social presence and cognitive load between video types with and without instructor–peer interaction and activity (with instructor–peer interaction and activity vs. without instructor–peer interaction and activity) in the 360 VR videos?

*H*3: The participants who viewed the 360 VR video with instructor–peer interaction and activity will have a higher social presence than those who viewed the video without instructor–peer interaction and activity.

*H*4: The participants who viewed the 360 VR video with instructor–peer interaction and activity will have a higher cognitive load than those who viewed the video without instructor–peer interaction and activity.

Justifications for hypotheses 3 & 4: RQ2 is designed to identify any differences among interaction types in the three 360 VR videos. We developed two types of videos, one with instructor–peer interaction and activity and the other without instructor–peer interaction and activity. H3 and H4 are our expectations of the second research question. The 360 VR video showing peers’ interaction and activity gives the users the feeling of being in a real classroom (Garrison and Cleveland-Innes, 2004). Additionally, in immersive environments, the presence or absence of vividness and interactivity has a significant impact on the sense of presence (Wallach et al., 2010). Whereas instructor interaction is often spontaneous and unintentional compared to peer interaction (Mehall, 2020). Peer interaction and activity in the instructional video might cause an unnecessarily higher cognitive load.

### 1.2 Theoretical frameworks

#### 1.2.1 Instructional 360 VR video

Notably, 360 VR can be an effective educational tool when students are training for high-risk or high-cost job roles that are difficult to practice in real-world settings. In addition, 360 VR videos allow users to perceive the virtual environment as an authentic scenario and they can explore the virtual situation freely (Southgate, 2018). The user is therefore no longer a passive spectator, but they are actively engaged in the learning experience they can explore deeply learning details embedded within the VR video (Roche et al., 2021). This also means that users can experience increased motivation, engagement, and presence of learning when in a real-world-like environment (Cheng and Tsai, 2019; Araiza-Alba et al., 2021). Furthermore, because 360 VR videos can be used as a tool for teaching and learning, they have similar cognitive learning effects with other tools—such as traditional videos and posters—although learners who experienced 360 VR videos report higher levels of interest and enjoyment than those trained using traditional media (Araiza-Alba et al., 2021). The positive feature of 360 VR is pervasive for educational purposes to provide situated learning experiences.

Due to common design processes, 360 VR videos often only provide visual stimuli. They are designed as a video experience, where the user’s only available interaction is the ability to move their heads and change their point-of-view (Pirker and Dengel, 2021). A freely selectable view of 360 VR videos mean that certain events can be easily overlooked when watching 360 VR videos (Ardisara and Fung, 2018). Therefore, users should continuously switch the focus of attention between the main target and other information in 360 VR videos (Lin et al., 2017). These additional requirements can detract from the overall user experience, leading users to miss some important events while they are still searching or exploring the scenario (Lin et al., 2017) and increasing perceived workload (Gold and Windscheid, 2020). Hence, additional design features are needed to enhance user-immersion and reduce unnecessary cognitive load. For this reason, some researchers have questioned the benefits of instructional 360 VR for content delivery, while conceding that the technique can increase learner engagement and interest (Lee et al., 2017; Hebbel-Seeger et al., 2021). Nevertheless, instructional 360 VR videos can favor learners’ understanding of theories or concepts related to a subject-specific topic (Ranieri et al., 2022). The immersive nature of 360 VR videos can improve student concentration on course content (Taubert et al., 2019). Hyttinen and Hatakka (2020) found that although 360 VR technology is suitable for lecturing, it is less suited to small group work or and promoting participation in discussions. Additionally, successfully implementing lectures using 360 VR videos requires the resolution of the inherent pedagogical and didactical challenges. For those instructors intending to use 360 VR videos in their teaching, they should consider appropriate teaching methods and strategies to promote learner engagement. The design considerations when creating 360 VR videos, such as the height of the 360 VR camera and the proximity and actions of people appearing in the videos, also influence learners’ viewing experiences and social presence (Saarinen et al., 2017; Keskinen et al., 2019). Through experiments, Keskinen et al. (2019) demonstrated that a camera height of approximately 150 cm and a distance of over 1 m between the camera and individuals provide the most comfortable viewing experience.

In sum, when developing 360 VR videos, it is essential to consider some factors that can affect the users’ social presence and cognitive load. Especially in instructional 360 VR videos for lecturing, the presence and interaction of the instructor and peers are important factors, because they create a learning atmosphere emulating a real classroom setting, ultimately affecting students’ learning experiences in the virtual environment.

#### 1.2.2 Social presence in instructional videos

Social presence is defined as a psychological phenomenon where someone is perceived as “real” during the communication process; it is the subjective feeling of being with other salient social actors in a technologically mediated space (Weidlich et al., 2018, p. 2146). In online learning, social–emotional aspects are essential factors during the design process. Social–emotional experiences should be differently designed in online learning from those in face-to-face learning. Unlike face-to-face environments that naturally provide rich social interactions, online learning demands unique instructional design strategies. It is challenging to imbue the online environment with meaningful social elements (Weidlich and Bastiaens, 2019) and to incorporate emotional, cognitive, and behavioral strategies that boost learner engagement and experience (Pentaraki and Burkholder, 2017). Online learning requires unique design approaches to emulate the rich social context of traditional face-to-face environments.

An increased perception of social presence can lead to increased task performance in multimedia learning environments (Schneider et al., 2022). While early studies on the predictors of social presence focused almost entirely on immersive qualities (e.g., visual representation, interactivity, haptic feedback, depth cues, audio quality, and display), more recent studies considered the impact of contextual and individual factors (e.g., personality/character traits of a virtual human, agency, physical proximity, task-type, social cues, identity cues, and psychological traits), perhaps as an acknowledgment of social presence as a subjective experience (Oh et al., 2018). Learners will engage in learning and then outperform their achievement by projecting individuals in the learning context in a trusting digital environment.

Within the virtual setting, the instructor’s role is important to enhance social presence. According to social agency theory, instructors who use social cues—such as tone of voice and speaking style—can promote social presence, stimulate cognitive processing, and improve learning outcomes (Mayer, 2014). In addition, when an instructor’s face is shown in a video lesson, it provides nonverbal communication cues, such as eye contact, gestures, and facial expressions; cues replicate the social aspect of human face-to-face interaction to the learners (Wang and Antonenko, 2017). Increased user social presence due to the inclusion of an instructor can result in deeper cognitive processing of learning content due to the activation of social interaction schema (Clark and Mayer, 2016). However, in a social learning environment, the role of peers cannot be overlooked. Social presence is heavily shaped through peer interaction (Garrison and Cleveland-Innes, 2004). According to the theory of social interaction, individuals not only perceive the existence of peers but also interact with peers through verbal and nonverbal means, which in turn leads to deeply cognition process and understanding for themselves (Zhu et al., 2022). Additionally, perceiving others may make learners change perspectives on the learning content or own learning processes (Schneider et al., 2022). From the results of previous studies, it is crucial to examine whether instructor or peers has more impact on a learner’s social presence in a 360 VR learning environment. The presence of peers may increase the sense of social presence rather than a single instructor’s influence on the learners.

#### 1.2.3 Cognitive load in 360 VR videos

When designing 360 VR videos for educational purposes, there should be consideration of the leaners’ ability to maintain concentration and the cognitive effort required to view the videos. In fact, 360 VR requires additional cognitive load for users to continue to focus on intended targets and refocus on another target (Lin et al., 2017). In addition, learners need to identify important information in the 360 VR videos, focus on it, and filter out relatively less important information. As a result, an instructional designer provides additional cues to mark important elements so users can adjust to the increased visual complexity, and make learners pay attention to specific points (Evens et al., 2022). Instructional considerations must be designed to keep learners attentive during 360 VR learning.

The presence of an instructor in instructional videos increases cognitive effort. Although social cues are intended to prime deeper processing in learning, a potential confounding factor is the role of cognitive load (Mayer, 2014). According to cognitive load theory, adding an image of the instructor may hinder attention engagement with the lecture’s content due to the split-attention effect (Ng and Przybyłek, 2021). In contrast, Henderson and Schroeder (2021) performed a systematic review of instructor presence in instructional videos, and did not find that an on-screen instructor increases extraneous processing and overall cognitive load. The researchers reported that further research is needed to better understand the effects of instructor’s gestures, facial expressions, and other components of the instructor’s appearance and their role within the learning environment. However, Hebbel-Seeger et al. (2021) found that an instructor-centered video is unsuitable for spherical projection due to its spatial setting, where only one viewing direction is used. For an instructor-centered lecture, the video needs to use the entire space, or focus on the content by combining the recorded lecture with additional visual elements. Conversely, studies have also shown that providing too much information, including interaction, in 360 VR videos can cause unnecessary cognitive loads on learners, so learners could not learn efficiently (Detyna and Dommett, 2021). The complexity of the video and excessive visual stimulation reduces the learner’s attention and can even lead to side effects such as VR motion sickness.

#### 1.2.4 Presence and interaction of instructor and peers

In general, greater interaction in an online learning environment increases the learner’s social presence (Horzum, 2017). However, interaction alone does not presume that one is engaged in a process if inquiry and cognitive presence exist (Picciano, 2002). In addition, the quality of interaction, not the quantity, is important to foster deep learning: high levels of interaction do not necessarily facilitate meaningful learning (Garrison and Cleveland-Innes, 2005). Therefore, interactions in VR may not always be purposeful, valuable, or contribute to student learning. Conversely, some interactions that do not directly relate to course content or learning objectives are without purpose and/or student benefit (Mehall, 2020). For this reason, the influence of interaction is different depending upon whom with the interaction, either peers or instructors for learners. The instructors are more concerned with fulfilling interaction needs (Hay et al., 2004). The instructor’s presence and interactions are planned to help learners meet the learning objectives, whereas peer interactions are often spontaneous and unintentional (Mehall, 2020). The primary reason for this differentiation by peers and instructors is that peers and instructors have influenced learners.

In online courses, interaction with instructors has a much larger effect than interaction with peers on satisfaction and perceived learning (Swan, 2001). The perceived presence of instructors may be a more influential factor in determining student satisfaction than the perceived presence of peers (Swan, 2001; Hay et al., 2004; Swan and Shih, 2005; Lowenthal and Dunlap, 2018). Similarly, Martin and Bolliger (2018) found that learner–instructor interaction is the most important among Moore’s three types of interactions: learner–instructor, learner–content, and learner–learner (Moore, 1989). Instructors can improve student engagement and learning by providing a variety of communication channels, support, encouragement, and timely feedback (Martin and Bolliger, 2018). Nevertheless, peer presence is an important factor to predict learners’ social presence. Swan and Shih (2005) found that the correlation between the perceived presence of instructors and perceived interaction lost significance when its relationship with the perceived presence of peers was controlled for, while the relationship between the perceived presence of peers and perceived interaction remained significant. This finding indicates that peer presence alone influences students’ perceptions of interactivity during course discussions.

Perceiving peer presence through modeling or observation can induce effectiveness when using digital materials, even if there are no “real” social interactions (Bandura, 1986). For example, many learners report feelings of intimidation when peers appear to have a deep understanding of the concepts being discussed (Cleveland-Innes et al., 2007). Moreover, some learners feel safe when they can share concerns and realize reassuringly that others shared similar worries (Peacock and Hooper, 2007). Thus, the instructor’s presence and interaction are normally intentional and have a significant impact on improving learners’ social presence and their learning (Swan, 2001; Hay et al., 2004; Swan and Shih, 2005; Lowenthal and Dunlap, 2018; Martin and Bolliger, 2018). However, learners tend to perceive the effect of peer presence and interaction differently depending on their individual learning styles or the course content (Swan and Shih, 2005; Mehall, 2020; Zhu et al., 2022). Therefore, when designing instructional 360 VR videos, the presence and interaction of the instructor should be considered first, but the presence and interaction of peers also needs to be carefully designed according to the purpose of the video.

## 2 Methods

### 2.1 Participants

A total of 48 college students (men = 19, women = 29) at a university located in a southwestern South Korean city participated in the experiment. The average age was 22.45 years (SD = 1.52). Among the 48 students, 11 were freshmen (22.9%), 18 were sophomores (37.5%), 13 were juniors (27.1%), and 6 were seniors (12.5%). Participants were randomly assigned to one of the three groups described above and they watched the video matched to the type of presence and interaction to their group.

### 2.2 Research design

A preplanned contrast analysis was employed to compare the three groups. We produced the three types of videos and assigned the participants randomly to each group. The participants were assigned into one of the three experimental settings, and they completed the surveys of social presence and cognitive load.

We set two components of video design; person featured in videos (instructor-only, instructor and peers) and instructor–peer interaction and activity (with instructor–peer interaction and activity, without instructor–peer interaction and activity). We designed the videos with the combinations of two components. Because it is impossible for the instructor to have peer interaction alone, “instructor-only × with instructor–peer interaction and activity” combination was excluded. Hence, we used three combinations: (1) instructor-only × without instructor–peer interaction and activity, (2) instructor and peers × without instructor–peer interaction and activity, and (3) instructor and peers × with instructor–peer interaction and activity. Each combination was matched to one of the three lecture video groups: (G1) instructor-only video, (G2) classroom lecture video, and (G3) classroom lecture and activity video. To determine which design is the most effective and practical among 360 VR videos for educational purposes, we measured participants’ social presence and cognitive load.

The research design of this study assumed the planned comparison to examine the specific effect of the independent variables. RQ1 addressed the effect of the person featured in 360 VR videos, so we compared G1 and G2 + G3 (planned comparison 1). Additionally, RQ2 evaluated the effect of instructor–peer interaction and activity in 360 VR videos, so we compared G1 + G2 and G3 (planned comparison 2). The contrast coefficient used for planned comparison 1 was as follows: ψ(G1) = −2, ψ(G2) = 1, ψ(G3) = 1, and in comparison plan 2 it was: ψ(G1) = 1, ψ(G2) = 1, ψ(G3) = −2.

### 2.3 Learning materials

The 360 VR videos were filmed in a classroom to ensure that only essential elements for the learning content—excluding people, tools, and materials—appeared. Unrelated individuals or objects could unnecessarily burden learners cognitively and disrupt their concentration. In all conditions, the instructor, a man in his mid-30s, remained consistent, as did the electric drills, woods, and tables used.

The content of the videos was the instructor’s classroom-based lecture, captured by a 360 VR camera, Insta 360° Pro2 (Insta360, 2024). Three types of 360 VR videos were designed and created for the instructional goals of basic carpentry practice with an electric drill as follows: (1) to learn the name of each component of an electric drill, (2) to learn how to use an electric drill when driving nails into wood, and (3) to understand how to prevent injuries that may occur when using an electrical drill. The target audience of the videos was university students who have no experience of carpentry practice. The videos included one instructor’s visual demonstration and verbal explanation about common practical steps when using an electric drill, and the video run-time was approximately 6 mins.

**Figure 1:**
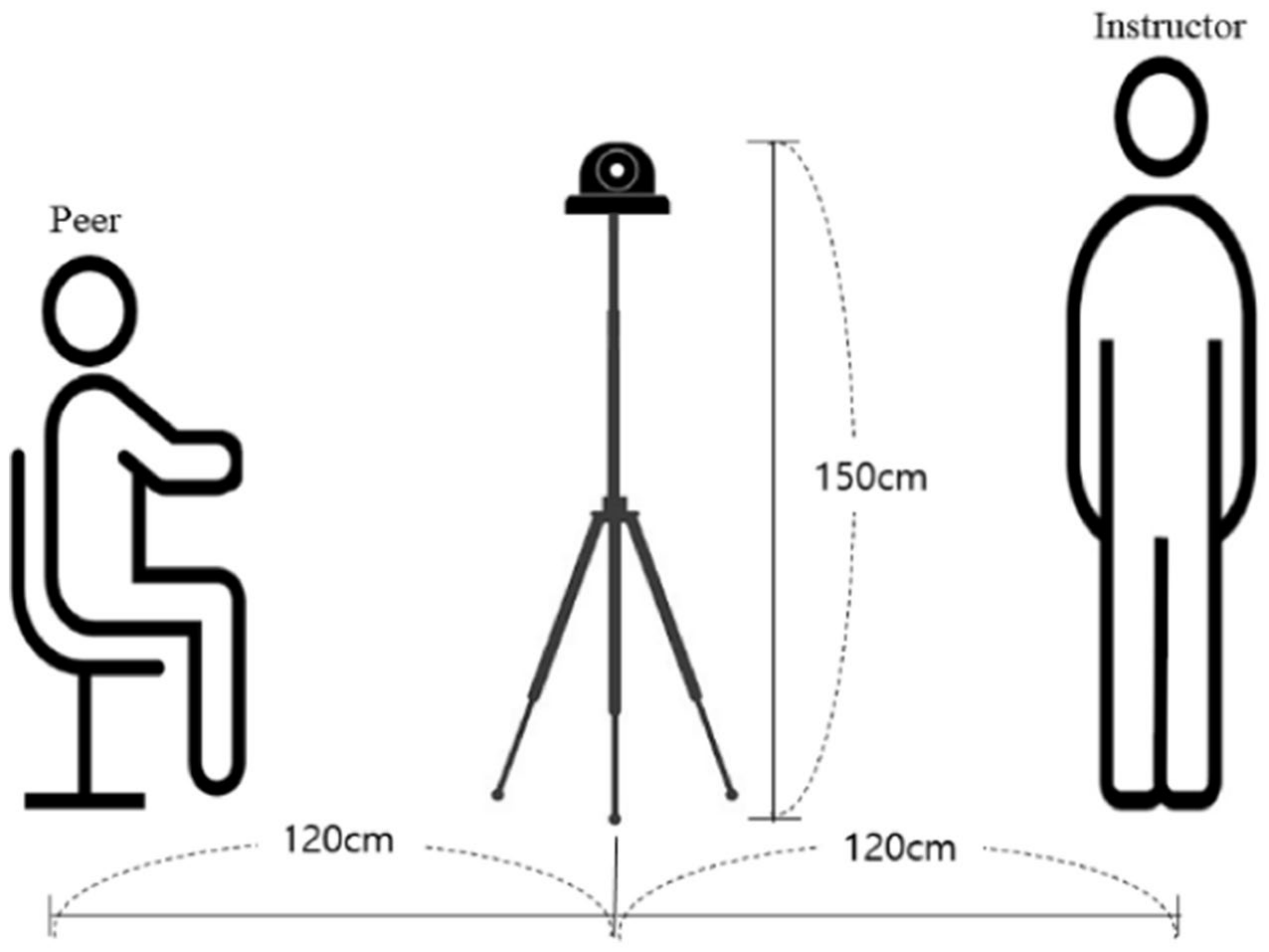
The camera height and distance from the viewer.

When filming the videos, the 360 VR camera was placed in the center of the classroom, and the instructor and/or peers were located around the camera. The position of the camera lens would be the ultimate viewpoint of the participant watching the 360 VR video. As shown in Figure 1, the height of the camera was 150 cm (4 ft. 11in), from which the viewers feel comfortable, regardless of their true height (Keskinen et al., 2019). In addition, the distance between the camera and the person featured was about 120 cm (3 ft. 11in), which is an appropriate distance so that participants do not feel burdened or disturbed when viewing the video (Saarinen et al., 2017). The size of video file was 7,680 mm × 4,320 mm, and the frame per second rate was 30.

### 2.4 Types of videos

**Figure 2:**
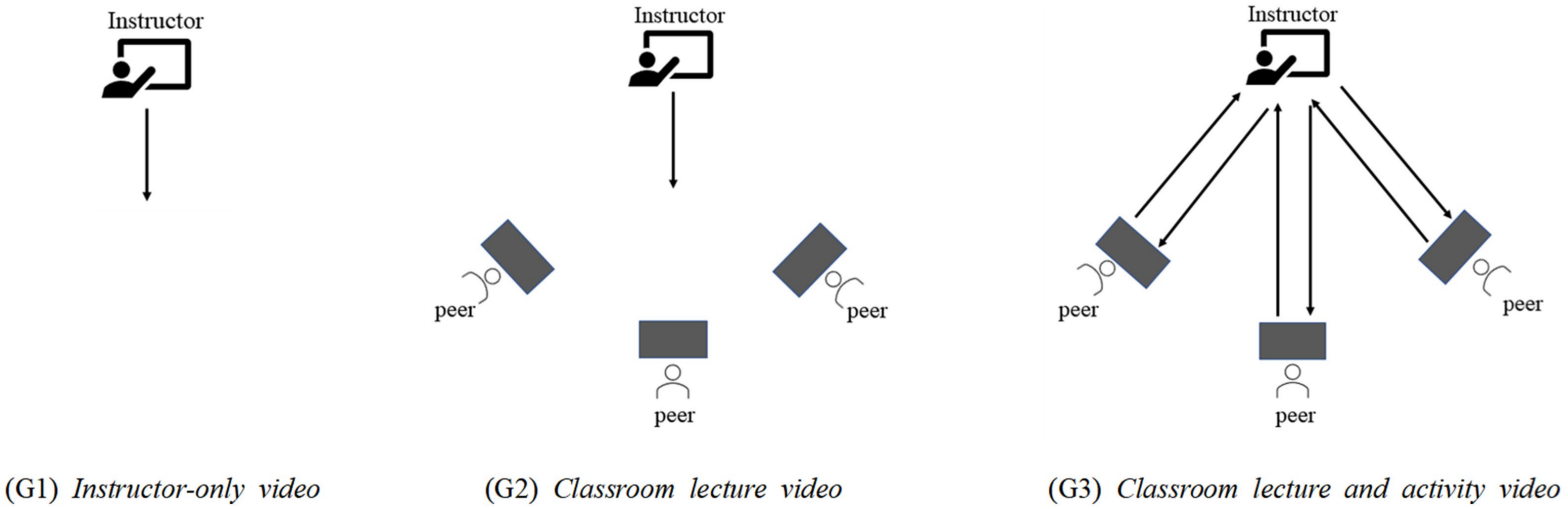
The three types of 360-degree VR videos. The arrows indicate the interaction and direction of both verbal and non-verbal interactions of the instructor and/or peers.

**Figure 3:**
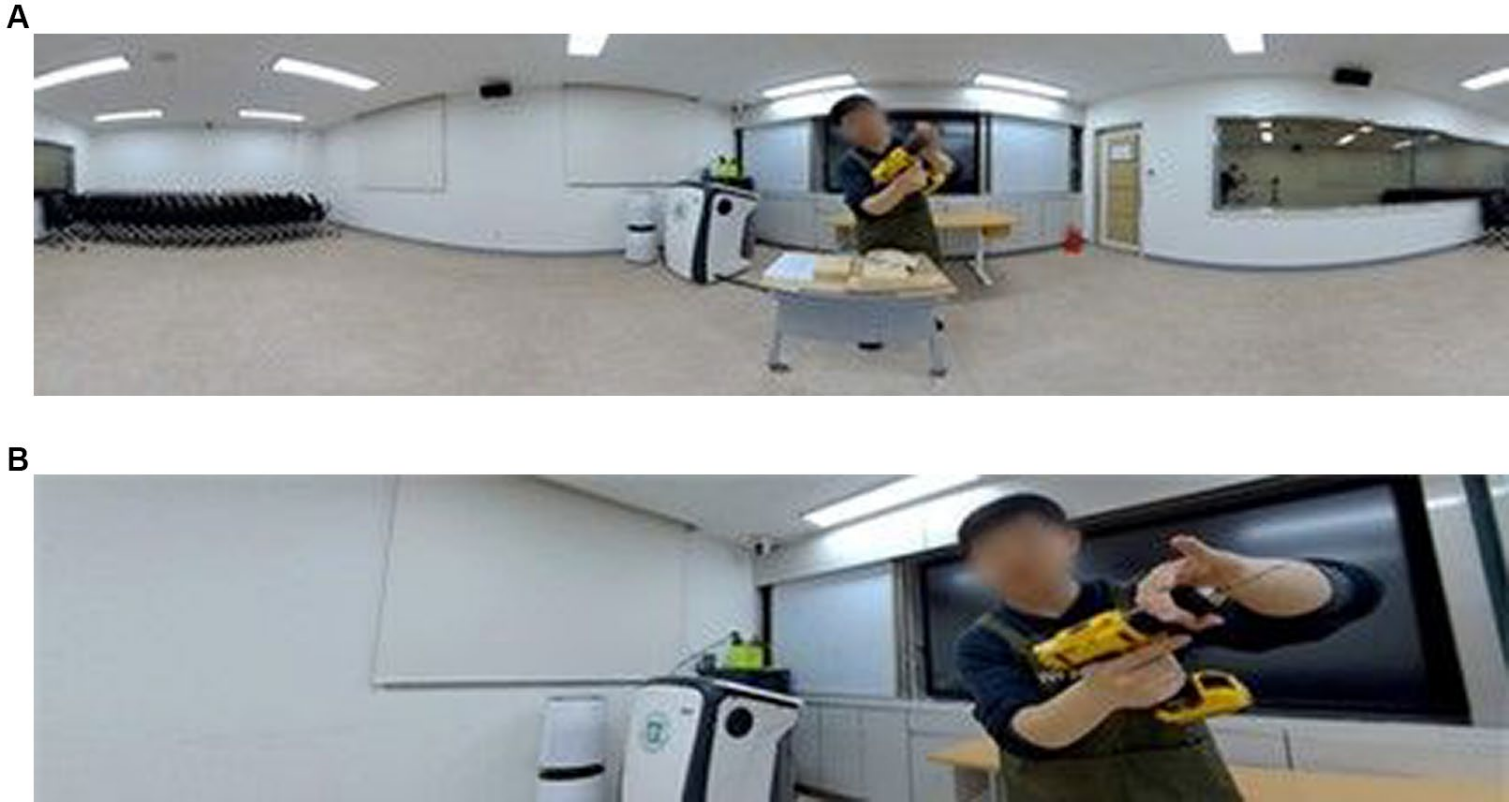
Screenshots of the instructor-only video. **(A)** full view; **(B)** participant view.

The three types of 360 VR videos, as shown in Figure 2, were as follows: (G1) Instructor-only video, (G2) Classroom lecture video and (G3) Classroom lecture and activity video. In G1, as shown in Figure 3, the video only shows the instructor who gave the lecture. All participants depicted in the figures of this study have provided their informed consent as documented by signed consent forms. The instructor explained and demonstrated the learning contents while directly looking at the 360 VR camera, as if the camera was a student in the classroom. From a participant’s viewpoint, the instructor continued to talk while looking only at them. Therefore, from their point-of-view, there were no others in the class and participants might feel as if only themselves and the instructor were in the virtual classroom.

**Figure 4:**
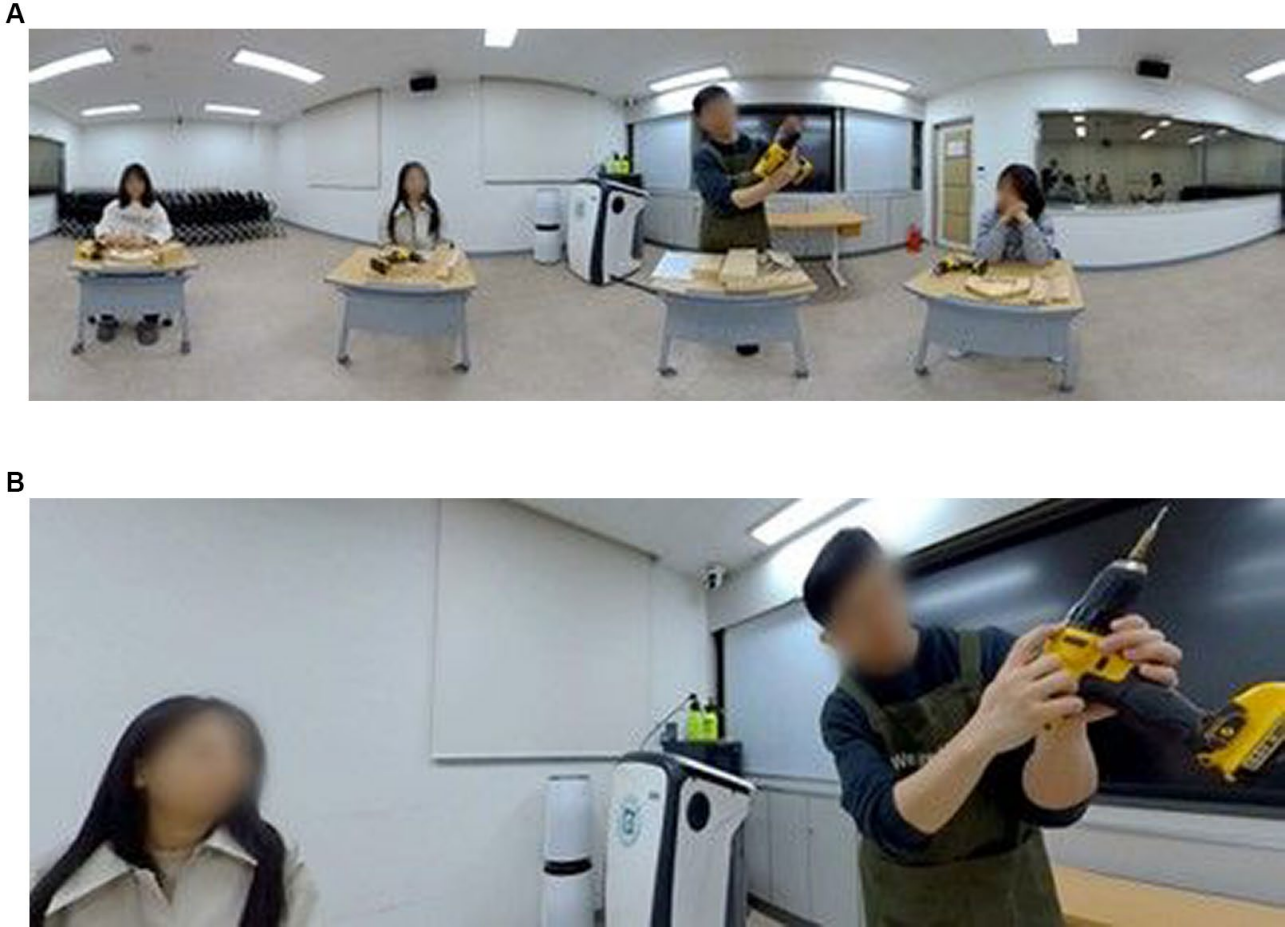
Screenshots of the classroom lecture video. **(A)** full view; **(B)** participant view.

In G2, as shown in Figure 4, the video showed both the instructor and other students in the classroom. However, there were no direct verbal or non-verbal interactions between the instructor and the students in the video. From the participant’s viewpoint, the instructor talked directly to the participant. In this case, the participant may feel that there were other students that attended the lesson, and were listening to the instructor, even if the instructor did not focus on the other students. Using the VR headset, participants could change their point-of-view whenever they wanted, and could choose to look at the instructor or their peers, but the other students did not perform any learning activities, they only listened to the instructor’s explanation and watching his demonstration.

**Figure 5:**
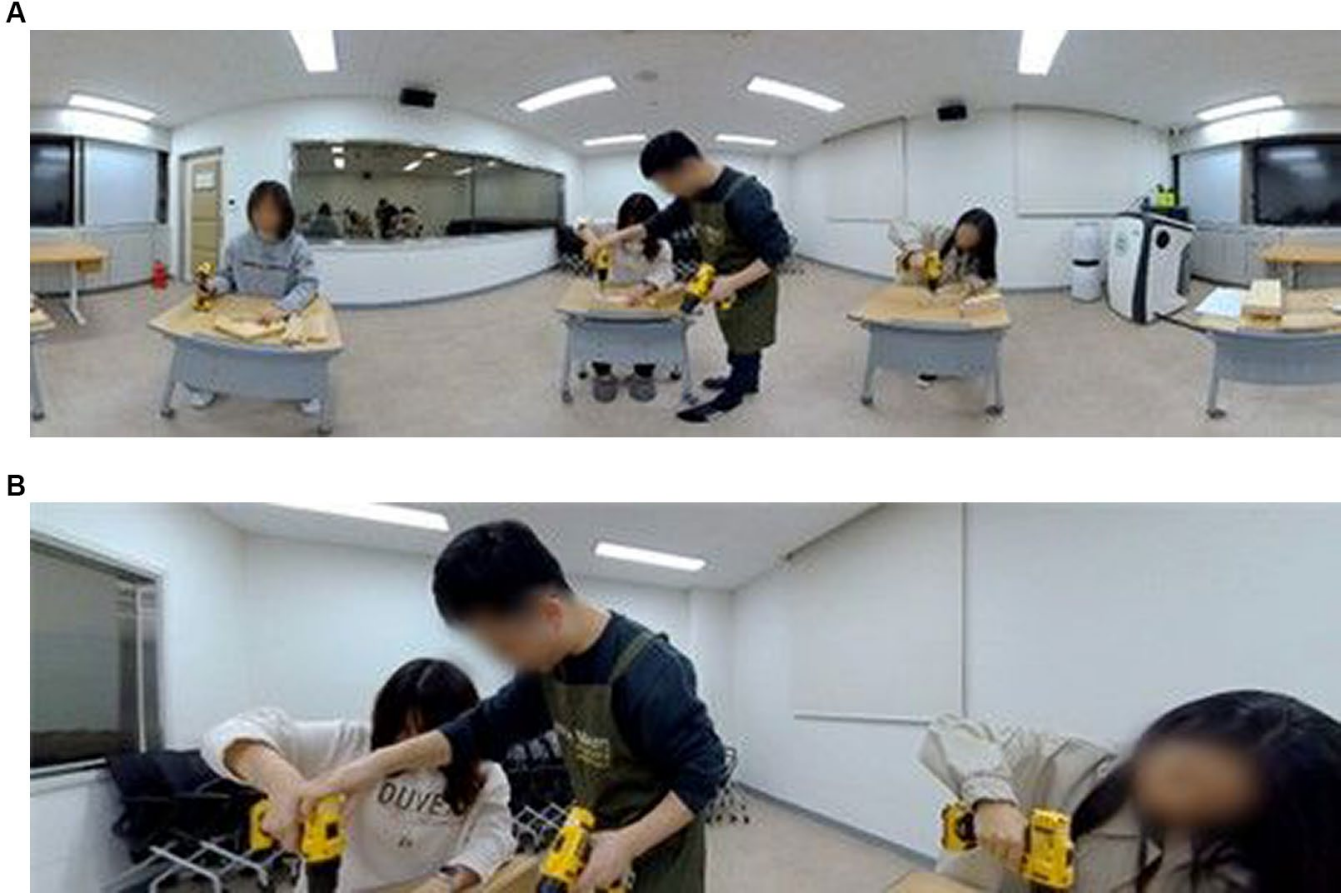
Screenshots of classroom lecture and activity video. **(A)** full view; **(B)** participant view.

In G3, as shown in Figure 5, the video showed both the instructor and other students in the classroom. Additionally, the lecture included active interaction sections, such as questions and answers, guidance, and practice activities with the instructor and students. These verbal and non-verbal interactions did not appear in G2. The instructor only occasionally glanced at the camera, and their main focus was the other students in the classroom. It was possible for participants to see the instructor’s explanations and demonstrations, as well as their peers’ learning activities, by altering their viewpoint using the VR headset.

### 2.5 Measurement

#### 2.5.1 Social presence

To evaluate the participants’ social presence while watching the 360 VR safety lesson video, the participants completed a questionnaire developed by Weidlich and Bastiaens (2019), after watching the lesson. This questionnaire consisted of 10 questions and its internal consistency was strong (α = 0.90). The examples of survey items were as follows: “In this learning environment, it feels as if we are a face-to-face group”; “In this learning environment, it feels as if all my fellow students are ‘real’ physical persons”; “In this learning environment, I imagine that I really can ‘see’ my fellow students in front of me.”

#### 2.5.2 Cognitive load

To evaluate the participants’ cognitive load while watching the 360 VR video, participants completed a questionnaire developed by Ryu and Yim (2009) after the lesson. The questionnaire had five subscales of cognitive load including task demand, mental effort, perceived task difficulty, self-evaluation, and material design. The questionnaire consisted of 20 items, with four items for each subscale. Additionally, the internal consistency subscales were good (0.57≤α≤.84). We provided the participants with a questionnaire translated into Korean. The examples of survey items are as follows: “I felt spent after the task [task demand]”; “I focused on the task to be performed [mental effort]”; “The difficulty of the task was high [perceived task difficulty]”; “I think that successfully understood the learning material [self-evaluation].”

#### 2.5.3 Procedures

The purpose of the study and the data collection procedure were explained before the experiment, and participants provided informed consent before the study began. Each participant wore an HMD and viewed the safety training lesson as assigned to their group. Participants were informed that they may stop the experiment at any time if they felt abnormal symptoms such as dizziness or cybersickness.

#### 2.5.4 Data analysis

The test results of social presence and cognitive load collected from the participants were analyzed using SPSS. Analysis of variance (ANOVA) was employed to analyze participants’ social presence. Also, a multivariate analysis of variance (MANOVA) was conducted to examine the different effects among and within groups. The process of verifying whether the assumptions, such as multivariate normality and homogeneity of variance–covariance matrices were met to ensure the validity of the MANOVA test results was included. The group effect of the 360 VR video types was calculated by overall group means using the F-ratio and the significant value. Two planned comparisons were then conducted. The first planned comparison was conducted to test the effect of the person featured; instructor-only (G1) vs. instructor and peer (G2 + G3). Additionally, the second planned comparison was conducted to test the effect of instructor–peer interaction and activity; without instructor–peer interaction and activity (G1 + G2) vs. with instructor–peer interaction and activity (G3).

## 3 Results

### 3.1 Social presence

**Figure 6:**
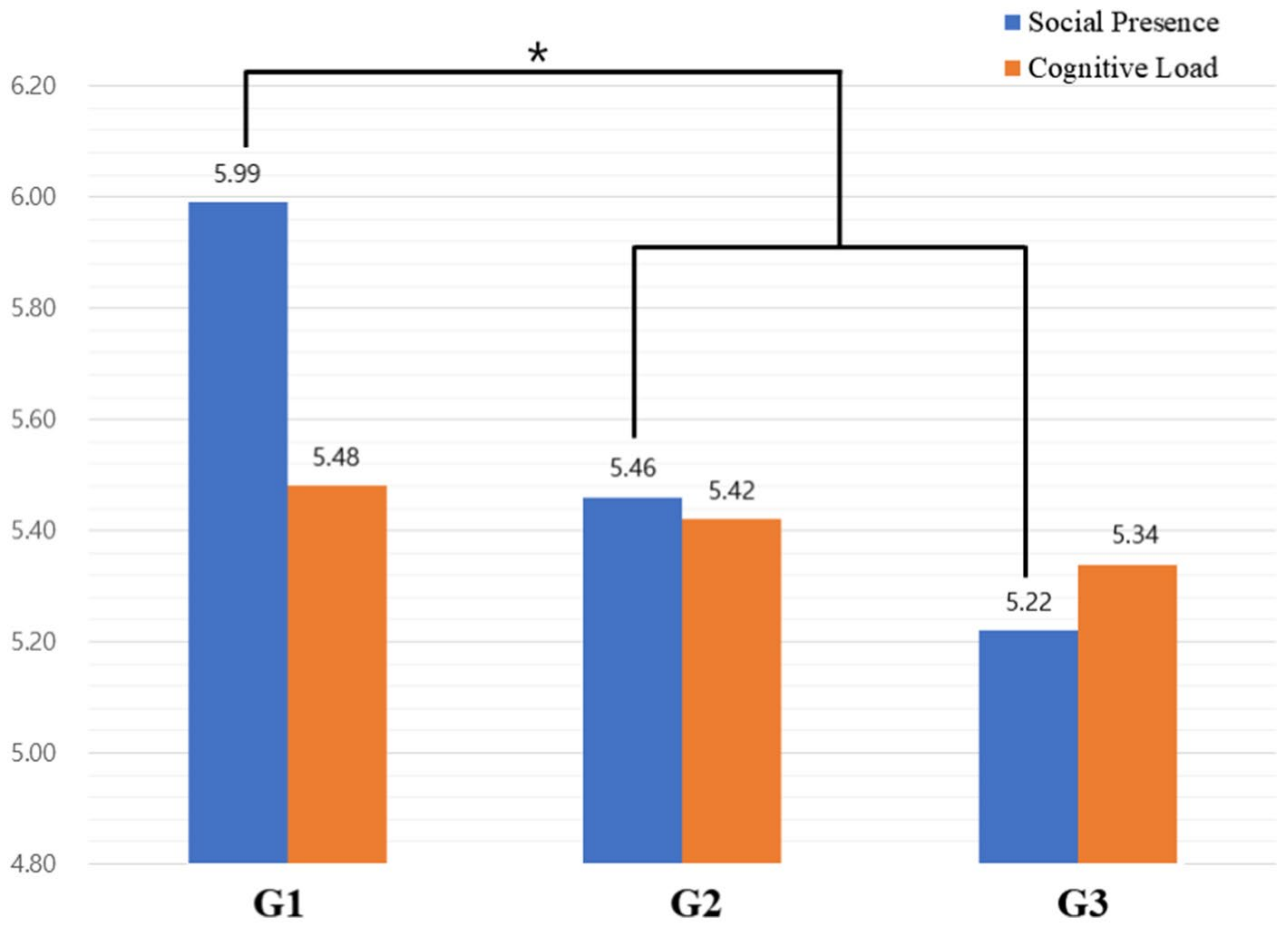
Mean Plot of Social Presence. *N* = 48. G1: Instructor-only video, G2: Classroom lecture video, G3: Classroom lecture and activity video.

Table 1 shows the descriptive statistical results of social presence scores by group. As a result of Levene’s test, error variances were the same (*p* = 0.714). ANOVA analysis found that there were no between-group effects [*F* (2, 45) = 2.41, *p* = 0.102]. Specifically, a planned comparison was conducted to determine differences in social presence among videos G1–G3. The first comparison was to confirm whether there are differences between the presence of peers. The results of the first planned comparison showed that 360 VR video without peers (G1) scored significantly higher as compared to G2 and G3 [*t* (45) = −2.09, *p* = 0.042] as shown in Figure 6. The second comparison examined the difference with and without instructor–peer interaction and learning activity. In this comparison, there was no difference in social presence [*t* (45) = 1.62, *p* = 0.112].

**Table 1:** Mean and standard deviation of social presence.

Group category		G1 (*n* = 16)	G2 (*n* = 16)	G3 (*n* = 16)	Total (*N* = 48)
		*M (SD)*	*M (SD)*	*M (SD)*	*M (SD)*
Social presence		5.99 (0.88)	5.46 (1.08)	5.22 (1.07)	5.55 (1.04)
Cognitive Load	Task Demand	2.91 (1.63)	2.75 (1.70)	2.02 (1.19)	2.56 (1.54)
	Mental Effort	5.50 (1.43)	5.67 (1.23)	6.08 (0.90)	5.75 (1.21)
	Perceived Task Difficulty	6.16 (0.81)	6.30 (0.75)	6.27 (0.75)	6.24 (0.81)
	Self-Evaluation	6.38 (0.59)	6.33 (0.86)	6.25 (0.65)	6.32 (0.69)

*N* = 48. G1: Instructor-only video, G2: Classroom lecture video, G3: Classroom lecture and activity video.

### 3.2 Cognitive load

Table 1 shows the descriptive statistical results of cognitive load scores by group. Cognitive load was classified into five subscales: task demand, mental effort, perceived task difficulty, self-evaluation, and material design. Box’s M test found that the assumption of equality of covariance was satisfied (*p* = 0.305) and Wilks’ lambda value was also satisfied (*p* = 0.183). As a result of Levene’s test, error variances were the same for all subscales (*p* > 0.05) and there were no between-group effects (*p* > 0.05). The second comparison to see the difference with and without peer interaction result in no difference in social presence [*t* (45) = 1.62, *p* = 0.112].

A planned contrasts test was conducted to find out the difference in cognitive load by the characteristics of 360 VR videos. The first contrast was to confirm whether there are differences between the presence of peers. The results of the first planned contrast showed that there was no difference in all subscales of cognitive load; task demand [*t* (45) = −1.12, *p* = 0.267], mental effort [*t* (45) = 1.01, *p* = 0.317], perceived task difficulty [*t* (45) = 0.50, *p* = 0.623], self- evaluation [*t* (45) = −0.40, *p* = 0.695], and material design 0[*t* (45) = −1.89, *p* = 0.065]. The second contrast found no difference among all subscales; task demand [*t* (45) = 1.75, *p* = 0.088], mental effort [*t* (45) = −1.33, *p* = 0.191], perceived task difficulty [*t* (45) = −0.16, *p* = 0.878], self-evaluation [*t* (45) = 0.47, *p* = 0.643], and material design [*t* (45) = 0.79, *p* = 0.433].

## 4 Discussion and conclusion

The purpose of this study was to examine the effects of instructor and peers’ presence and interaction in 360 VR instructional videos on students perceived social presence and cognitive load. We developed three types of 360 VR videos. After watching one type of video, participants completed questionnaires that assessed their social presence and cognitive load. The data was used to assess the effects of each type of 360 VR video.

In this study, the participants reported the highest level of social presence in the instructor-only video (G1). This is probably due to the nature of the video format. Only one-instructor showed on the video, and the instructor’s explanation was only toward the participants. From the viewer’s perspective, the participant of G1 would perceive direct interaction with the instructor. The instructor continued to look into camera lens, and therefore lectured to the participant. These social cues used by the instructor enhance users’ social presence (Swan, 2001; Hay et al., 2004; Swan and Shih, 2005; Lowenthal and Dunlap, 2018; Martin and Bolliger, 2018). Notably however, peer presence impeded establishing participants’ social presence. This result is inconsistent with previous studies that suggest that peer presence promotes learners’ social presence (Cleveland-Innes et al., 2007; Peacock and Hooper, 2007). It is possible that the participants found the peer presence less useful than the lesson content or the instructor’s explanations in the 360 VR video (Zhu et al., 2022). When watching the 360 VR videos, participants could change their point-of-view freely, so it was possible to only view the instructor, therefore some participants might not have looked around and watched their peers. Participants not moving their head to look at their peers during the lecture may be the reason that there was no difference in participants’ cognitive load among all video types (G1–G3), including comparisons of peer presence and peer interaction. We can infer that all participants paid similar amounts of mental efforts for learning in all groups. Furthermore, participants’ learning was unaffected by the presence of peers or peer interactions in the videos. Participants in G1, based only on the instructor’s explanations, understood the content as well as their counterparts in G2 and G3. This supports findings reported in other studies based on instructional videos, where learners perceived interaction with instructors more influential and valuable than interaction with peers on perceived learning (Swan, 2001; Hay et al., 2004; Swan and Shih, 2005; Lowenthal and Dunlap, 2018).

The findings of this study reaffirm those in the literature that in non-face-to-face learning, the instructor’s presence might be more critical than peer presence and peer interaction. Instructor interaction is decisive for predicting delivery effectiveness in online courses and achieving learning success (Hay et al., 2004; Belair, 2012), while peer interaction may not directly contribute to learners’ success (Keaton and Gilbert, 2020). Although a previous study found that 360 VR is an effective learning tool when presenting the entire spherical space (Pirker and Dengel, 2021), this study showed that only including the instructor and their interactions can be equally effective. This result may be due to the immersive nature of the 360 VR videos, which facilitates learners’ concentration on content (Taubert et al., 2019). There was no significant difference in cognitive load among the groups. No significant impact may be because participants in this study did not have to find the focus of the video, as the instructor was easy to locate in the virtual classroom. The lack of visual clues and additional guidance embedded in the 360 VR videos may have prevented learners from missing important content.

Some limitations of the study should be noted. It is difficult to generalize the results of this study because the teaching materials used were limited to safety training for woodworking and the use of an electric drill. Moreover, the peer–peer interactions in the videos were very simple. Further 360 VR research that includes meaningful peer–peer interactions in other settings is needed. Indeed, it is essential to acknowledge that the primary focus of this study was on measuring perceptual changes in engagement during learning rather than on direct measures of learning achievement. While engagement is a critical precursor to learning, it does not necessarily predict the acquisition of knowledge or skills directly. Consequently, this study’s findings primarily indicate shifts in learner engagement and may not directly reflect changes in academic achievement or proficiency. These limitations suggest the need for further research that explicitly assesses the impact of non-face-to-face learning environments on measurable learning outcomes.

In conclusion, this study confirms the effects of the instructor and peer presence and interactions on learners’ social presence and cognitive load. When only an instructor is present and instructor–learner interaction can be viewed, learners report high levels of social presence. Lower levels of social presence are reported when learners only view their class peers and no meaningful peer–peer or instructor–peer interactions are included in the video. Therefore, when developing 360 VR videos, educators should design the virtual location primarily considering the user’s view, and focus on the presence and interactions of instructors rather than peers. Except in the case of discussion or cooperative learning where interaction between peers is essential and meaningful, there is no need to include the presence or interaction of peers into the video.

Learners who use educational 360 VR videos tend to follow the lesson based on social clues, such as the instructor’s voice and their facial expressions. These features allow learners to take part in the virtual lecture without reporting excessive cognitive loads. However, long video run-times may increase boredom levels in instructor-centered lectures when there is no peer–peer interactions. In these cases, visual cues are recommended to maintain students’ attention to the instructor and the content. The inclusion of peer-to-peer interaction should be chosen carefully, taking into account the content and context of the learning.

## Data availability statement

The raw data supporting the conclusions of this article will be made available by the authors, without undue reservation.

## Ethics statement

The studies involving humans were approved by Chonnam National University Institutional Review Board. The studies were conducted in accordance with the local legislation and institutional requirements. The participants provided their written informed consent to participate in this study. Written informed consent was obtained from the individual(s) for the publication of any potentially identifiable images or data included in this article.

## Author contributions

SK: Writing – original draft, Writing – review & editing. SP: Writing – review & editing. JR: Writing – review & editing. JK: Writing – original draft. IK: Validation, Writing – review & editing.

## References

[ref1006] Alraimi K. M. Zo H. Ciganek A. P. (2015). Understanding the MOOCs continuance: The role of openness and reputation. Computers & Education 80, 28–38. doi: 10.1016/j.compedu.2014.08.006

[ref1] Andel S. A. de Vreede T. Spector P. E. Padmanabhan B. Singh V. K. De Vreede G. J. (2020). Do social features help in video-centric online learning platforms? A social presence perspective. Comput. Hum. Behav. 113:106505. doi: 10.1016/j.chb.2020.106505

[ref2] Araiza-Alba P. Keane T. Matthews B. Simpson K. Strugnell G. Chen W. S.. (2021). The potential of 360°-degree virtual reality videos to teach water-safety skills to children. Comput. Educ. 163:104096. doi: 10.1016/j.compedu.2020.104096

[ref3] Ardisara A. Fung F. M. (2018). Integrating 360 videos in an undergraduate chemistry laboratory course. J. Chem. Educ. 95, 1881–1884. doi: 10.1021/acs.jchemed.8b00143

[ref5] Bandura A. (1986). The explanatory and predictive scope of self-efficacy theory. J. Soc. Clin. Psychol. 4, 359–373. doi: 10.1521/jscp.1986.4.3.359

[ref1002] Beege M. Krieglstein F. Arnold C. (2012). How instructors influence learning with instructional videos-The importance of professional appearance and communication. Computers & Education 185:104531.

[ref1003] Beege M. Nebel S. Schneider S. Rey G. D. (2023). The effect of microlevel and macrolevel signaling on learning with 360° videos. Applied Cognitive Psychology 37, 232–246. doi: 10.1002/acp.4023

[ref6] Belair M. (2012). The investigation of virtual school communications. TechTrends 56, 26–33. doi: 10.1007/s11528-012-0584-2

[ref1004] Boling E. C. Hough M. Krinsky H. Saleem H. Stevens M. (2012). Cutting the distance in distance education: Perspectives on what promotes positive, online learning experiences. The Internet and Higher Education 15, 118–126. doi: 10.1016/j.iheduc.2011.11.006

[ref8] Borup J. West R. E. Graham C. R. (2012). Improving online social presence through asynchronous video. Internet High. Educ. 15, 195–203. doi: 10.1016/j.iheduc.2011.11.001

[ref1007] Breslow L. Pritchard D. E. DeBoer J. Stump G. S. Ho A. D. Seaton D. T. (2013). Studying learning in the worldwide classroom research into edX’s first MOOC. Research & Practice in Assessment 8, 13–25. Retrieved from https://files.eric.ed.gov/fulltext/EJ1062850.pdf

[ref1008] Cesari V. Galgani B. Gemignani A. Menicucci D. (2021). Enhancing qualities of consciousness during online learning via multisensory interactions. Behavioral Sciences 11:57. doi: 10.3390/bs11050057 33919379 PMC8143304

[ref9] Cheng K. H. Tsai C. C. (2019). A case study of immersive virtual field trips in an elementary classroom: students’ learning experience and teacher-student interaction behaviors. Comput. Educ. 140:103600. doi: 10.1016/j.compedu.2019.103600

[ref1009] Chi M. T. Kang S. Yaghmourian D. L. (2017). Why students learn more from dialogue-than monologue-videos: Analyses of peer interactions. Journal of the Learning Sciences 26, 10–50. doi: 10.1080/10508406.2016.1204546

[ref10] Clark R. C. Mayer R. E. (2016). E-learning and the science of instruction: Proven guidelines for consumers and designers of multimedia learning. New Jersey, NJ: John Wiley & Sons.

[ref11] Cleveland-Innes M. Garrison R. Kinsel E. (2007). Role adjustment for learners in an online community of inquiry: identifying the challenges of incoming online learners. Int. J. Web-Based Learn. Teach. Technol. (IJWLTT) 2, 1–16. doi: 10.4018/jwltt.2007010101

[ref12] Dede C. Grotzer T. A. Kamarainen A. Metcalf S. J. (2017). “Virtual reality as an immersive medium for authentic simulations” in Virtual, augmented, and mixed realities in education. eds. Liu D. Dede C. Huang R. Richards J. (Singapore: Smart Computing and Intelligence. Springer).

[ref13] Detyna M. Dommett E. (2021). An investigation into digital tools for lecture engagement: a feasibility study. Compass: J. Learn. Teach. 14, 1–19. Available at: https://journals.gre.ac.uk/index.php/compass/article/download/1114/pdf

[ref14] Evens M. Empsen M. Hustinx W. (2022). A literature review on 360-degree video as an educational tool: towards design guidelines. J. Comput. Educ. 10, 325–375. doi: 10.1007/s40692-022-00233-z

[ref15] Garrison D. R. Cleveland-Innes M. (2004). Critical factors in student satisfaction and success: facilitating student role adjustment in online communities of inquiry. Elements of quality online education: Into the mainstream 5, 29–38. Available at: https://urn.kb.se/resolve?urn=urn:nbn:se:kth:diva-71040

[ref16] Garrison D. R. Cleveland-Innes M. (2005). Facilitating cognitive presence in online learning: interaction is not enough. Am. J. Dist. Educ. 19, 133–148. doi: 10.1207/s15389286ajde1903_2

[ref17] Gold B. Windscheid J. (2020). Observing 360-degree classroom videos–effects of video type on presence, emotions, workload, classroom observations, and ratings of teaching quality. Comput. Educ. 156:103960. doi: 10.1016/j.compedu.2020.103960

[ref18] Gunawardena C. N. Zittle F. J. (1997). Social presence as a predictor of satisfaction with a computer-mediated conferencing environment. Am. J. Dist. Educ. 11, 8–26. doi: 10.1080/08923649709526970

[ref19] Hay A. Hodgkinson M. Peltier J. W. Drago W. A. (2004). Interaction and virtual learning. Strateg. Chang. 13, 193–204. doi: 10.1002/jsc.679

[ref20] Hebbel-Seeger A. Riehm P. Kopischke A. Baranovskaa M. (2021). LectureCast as 360 degree video: what impact do immersion and presence experience have on learning performance? Athens J. Educ. 8, 23–36. doi: 10.30958/aje.8-1-2

[ref21] Henderson M. L. Schroeder N. L. (2021). A systematic review of instructor presence in instructional videos: effects on learning and affect. Computers and Educ. Open 2:100059. doi: 10.1016/j.caeo.2021.100059

[ref22] Horzum M. B. (2017). Interaction, structure, social presence, and satisfaction in online learning. Eurasia J. Mathematics, Sci. Technol. Educ. 11, 505–512. doi: 10.12973/eurasia.2014.1324a

[ref24] Hyttinen M. Hatakka O. (2020). The challenges and opportunities of using 360-degree video technology in online lecturing: a case study in higher education business studies. Seminar 16:16. doi: 10.7577/seminar.2870

[ref25] Insta360. (2024). Insta360 Pro2. Available at: https://www.insta360.com/kr/product/insta360-pro2

[ref28] Keaton W. Gilbert A. (2020). Successful online learning: what does learner interaction with peers, instructors and parents look like? J. Online Learn. Res. 6, 129–154. Available at: https://www.learntechlib.org/primary/p/215616/

[ref29] Keskinen T. Mäkelä V. Kallioniemi P. Hakulinen J. Karhu J. Ronkainen K. (2019). The effect of camera height, actor behavior, and viewer position on the user experience of 360 videos. In *2019 IEEE conference on virtual reality and 3D user interfaces (VR)*. IEEE. 1–18. Available at: http://dice.newcastle.edu.au/DRS_6_2018.pdf

[ref1011] Lampropoulos G. Barkoukis V. Burden K. Anastasiadis T. (2021). 360-degree video in education: An overview and a comparative social media data analysis of the last decade. Smart Learning Environments 8:20. doi: 10.1186/s40561-021-00165-8

[ref30] Lee S. H. Sergueeva K. Catangui M. Kandaurova M. (2017). Assessing Google cardboard virtual reality as a content delivery system in business classrooms. J. Educ. Bus. 92, 153–160. doi: 10.1080/08832323.2017.1308308

[ref31] Lin Y. T. Liao Y. C. Teng S. Y. Chung Y. J. Chan L. Chen B. Y. (2017). Outside-in: visualizing out-of-sight regions-of-interest in a 360° video using spatial picture-in-picture previews. In *Proceedings of the 30th Annual ACM Symposium on User Interface Software and Technology* (pp. 255–265).

[ref32] Lowenthal P. R. Dunlap J. C. (2018). Investigating students’ perceptions of instructional strategies to establish social presence. Distance Educ. 39, 281–298. doi: 10.1080/01587919.2018.1476844

[ref1012] Makransky G. Petersen G. B. (2021). The cognitive affective model of immersive learning (CAMIL): A theoretical research-based model of learning in immersive virtual reality. Educational Psychology Review 33, 937–958. doi: 10.1007/s10648-020-09586-2

[ref33] Martin F. Bolliger D. U. (2018). Engagement matters: student perceptions on the importance of engagement strategies in the online learning environment. Online Learn. 22, 205–222. doi: 10.24059/olj.v22i1.1092

[ref34] Mayer R. E. (2014). Principles based on social cues in multimedia learning: personalization, voice, image, and embodiment principles. Cambridge handbook of multimedia learning 16, 345–370. doi: 10.1017/CBO9781139547369.017

[ref35] Mehall S. (2020). Purposeful interpersonal interaction in online learning: what is it and how is it measured? Online Learn. 24, 182–204. doi: 10.24059/olj.v24i1.2002

[ref36] Moore M. G. (1989). Three types of interaction. Am. J. Dist. Educ. 3, 1–7. doi: 10.1080/08923648909526659

[ref37] Ng Y. Y. Przybyłek A. (2021). Instructor presence in video lectures: preliminary findings from an online experiment. IEEE Access 9, 36485–36499. doi: 10.1109/ACCESS.2021.3058735

[ref38] Oh C. S. Bailenson J. N. Welch G. F. (2018). A systematic review of social presence: definition, antecedents, and implications. Front. Robo. AI 5:114. doi: 10.3389/frobt.2018.00114, PMID: 33500993 PMC7805699

[ref1005] Okita Y. Narita Y. Miyakita Y. Ohno M. Matsushita Y. Fukushima S.. (2012). IDH1/2 mutation is a prognostic marker for survival and predicts response to chemotherapy for grade II gliomas concomitantly treated with radiation therapy. International Journal of Oncology 41, 1325–1336. doi: 10.3892/ijo.2012.1564 22825915

[ref39] Peacock S. Hooper J. (2007). E-learning in physiotherapy education. Physiotherapy 93, 218–228. doi: 10.1016/j.physio.2006.11.009

[ref40] Pentaraki A. Burkholder G. J. (2017). Emerging evidence regarding the roles of emotional, Behavioural, and cognitive aspects of student engagement in the online classroom. European J. Open, Distance and E-Learn. 20, 1–21. doi: 10.1515/eurodl-2017-0001

[ref41] Picciano A. G. (2002). Beyond student perceptions: issues of interaction, presence, and performance in an online course. JALN 6, 21–40. Available at: https://pdfs.semanticscholar.org/bfdd/f2c4078b58aefd05b8ba7000aca1338f16d8.pdf

[ref1013] Pimentel D. Vinkers C. (2021). Copresence with virtual humans in mixed reality: The impact of contextual responsiveness on social perceptions. Frontiers in Robotics and AI 8:634520. doi: 10.3389/frobt.2021.634520 33912595 PMC8072477

[ref42] Pirker J. Dengel A. (2021). The potential of 360°° virtual reality videos and real VR for education: a literature review. IEEE Comput. Graph. Appl. 41, 76–89. doi: 10.1109/MCG.2021.3067999, PMID: 33755559

[ref43] Ranieri M. Luzzi D. Cuomo S. Bruni I. (2022). If and how do 360 videos fit into education settings? Results from a scoping review of empirical research. J. Comput. Assist. Learn. 38, 1199–1219. doi: 10.1111/jcal.12683

[ref45] Roche L. Kittel A. Cunningham I. Rolland C. (2021). 360 video integration in teacher education: a SWOT analysis. Front. Educ. 6:761176. doi: 10.3389/feduc.2021.761176

[ref46] Ryu J. H. Yim J. H. (2009). An exploratory validation for the constructs of cognitive load. J. Korean Association for Educ. Info. Media 15, 1–27. Available at: https://www.riss.kr/link?id=A76565358

[ref47] Saarinen S. Mäkelä V. Kallioniemi P. Hakulinen J. Turunen M. (2017). “Guidelines for designing interactive omnidirectional video applications” in Human-computer interaction – INTERACT 2017. INTERACT 2017. Lecture notes in computer science. eds. Bernhaupt R. Dalvi G. Joshi A. Balkrishan K., vol. 10516 (Cham: Springer).

[ref48] Schneider S. Beege M. Nebel S. (2022). The cognitive-affective-social theory of learning in digital environments (CASTLE). Educ. Psychol. Rev. 34, 1–38. doi: 10.1007/s10648-021-09626-5 34226808 PMC8242289

[ref49] Shadiev R. Yu J. Sintawati W. (2021). Exploring the impact of learning activities supported by 360-degree video technology on language learning, intercultural communicative competence development, and knowledge sharing. Front. Psychol. 12:766924. doi: 10.3389/fpsyg.2021.766924, PMID: 34899512 PMC8663917

[ref1014] Skuballa I. T. Xu K. M. Jarodzka H. (2019). The impact of co-actors on cognitive load: When the mere presence of others makes learning more difficult. Computers in Human Behavior 101, 30–41. doi: 10.1016/j.chb.2019.06.016

[ref1010] Snelson C. Hsu Y. C. (2020). Educational 360-degree videos in virtual reality: A scoping review of the emerging research. TechTrends 64, 404–412. doi: 10.1007/s11528-019-00474-3

[ref50] Southgate E. (2018). Immersive virtual reality, children and school education: a literature review for teachers. DICE Report Series 6, 1–18. Available at: http://dice.newcastle.edu.au/DRS_6_2018.pdf

[ref51] Swan K. (2001). Virtual interaction: design factors affecting student satisfaction and perceived learning in asynchronous online courses. Distance Educ. 22, 306–331. doi: 10.1080/0158791010220208

[ref52] Swan K. Shih L. F. (2005). On the nature and development of social presence in online course discussions. JALN 9, 115–136. Available at: https://pdfs.semanticscholar.org/ba90/92141bf9e535787ea7e912f066841bd7f67e.pdf

[ref53] Taubert M. Webber L. Hamilton T. Carr M. Harvey M. (2019). Virtual reality videos used in undergraduate palliative and oncology medical teaching: results of a pilot study. BMJ Support. Palliat. Care 9, 281–285. doi: 10.1136/bmjspcare-2018-001720, PMID: 30808627 PMC6817702

[ref54] Torres A. Carmichael C. Wang W. Paraskevakos M. Uribe-Quevedo A. Giles P. (2020). A 360° video editor framework for interactive training. In 2020 IEEE *8th international conference on serious games and applications for health (SeGAH)* (pp. 1–7). IEEE.

[ref55] Ulrich F. Helms N. H. Frandsen U. P. Rafn A. V. (2021). Learning effectiveness of 360 video: experiences from a controlled experiment in healthcare education. Interact. Learn. Environ. 29, 98–111. doi: 10.1080/10494820.2019.1579234

[ref1015] Wallach H. S. Safir M. P. Samana R. (2010). Personality variables and presence. Virtual Reality 14, 3–13. doi: 10.1007/s10055-009-0124-3

[ref56] Wang J. Antonenko P. D. (2017). Instructor presence in instructional video: effects on visual attention, recall, and perceived learning. Comput. Hum. Behav. 71, 79–89. doi: 10.1016/j.chb.2017.01.049

[ref57] Weidlich J. Bastiaens T. J. (2019). Designing sociable online learning environments and enhancing social presence: an affordance enrichment approach. Comput. Educ. 142:103622. doi: 10.1016/j.compedu.2019.103622

[ref1018] Weidlich J. Kreijns K. Rajagopal K. Bastiaens T. (2018). “What Social Presence is, what it isn’t, and how to measure it: A work in progress” in Proceedings of EdMedia: World Conference on Educational Media and Technology. eds. Bastiaens T. Van Braak J. Brown M. Cantoni L. Castro M. Christensen R. Amsterdam, Netherlands: Association for the Advancement of Computing in Education (AACE), 2142–2150. Available at: https://www.learntechlib.org/primary/p/184456/ (Accessed June 18, 2024).

[ref60] Zhu F. Yang J. Pi Z. (2022). Benefits of peer learning and learning by teaching for students learning through instructional videos. In 2022 *IEEE 2nd international conference on educational technology (ICET)* (pp. 96–100). IEEE.

